# Asymmetry of P1 and vertebral arteries is not related to basilar tip aneurysm development or rupture

**DOI:** 10.1007/s00701-020-04593-2

**Published:** 2020-10-06

**Authors:** Lan Li, Björn B. Hofmann, Igor Fischer, Daniel M. Donaldson, Adrian Engel, Cihat Karadag, Andreas Wetzel-Yalelis, Guilherme Santos Piedade, Hendrik-Jan Mijderwijk, Richard Bostelmann, Marius G. Kaschner, Sajjad Muhammad, Daniel Hänggi, Jan F. Cornelius, Athanasios K. Petridis

**Affiliations:** 1 Medical Faculty, Heinrich Heine University Duesseldorf, Duesseldorf, Germany; 2 Department of Neurosurgery, Medical Faculty, Heinrich Heine University Duesseldorf, Duesseldorf, Germany; 3 Department of Diagnostic and Interventional Radiology, Medical Faculty, Heinrich Heine University Duesseldorf, Duesseldorf, Germany

**Keywords:** Basilar aneurysm, Aneurysm development, Aneurysm rupture, Asymmetry of P1, Asymmetry of vertebral arteries, Aneurysm lobuli, Subarachnoid hemorrhage

## Abstract

**Objective:**

Lately, morphological parameters of the surrounding vasculature aside from aneurysm size, specific for the aneurysm location, e.g., posterior cerebral artery angle for basilar artery tip aneurysms, could be identified to correlate with the risk of rupture. We examined further image-based morphological parameters of the aneurysm surrounding vasculature that could correlate with the growth or the risk of rupture of basilar artery tip aneurysms.

**Methods:**

Data from 83 patients with basilar tip aneurysms (27 not ruptured; 56 ruptured) and 100 control patients were assessed (50 without aneurysms and 50 with aneurysms of the anterior circle of Willis). Anatomical parameters of the aneurysms were assessed and analyzed, as well as of the surrounding vasculature, namely the asymmetry of P1 and the vertebral arteries.

**Results:**

Patients with basilar tip aneurysm showed no significant increase in P1 or vertebral artery asymmetry compared with the control patients or patients with aneurysms of the anterior circulation, neither was there a significant difference in asymmetry between cases with ruptured and unruptured aneurysms. Furthermore, we observed no significant correlations between P1 asymmetry and the aneurysm size or number of lobuli in the aneurysms.

**Conclusion:**

We observed no significant difference in aneurysm size, rupture, or lobulation associated with P1 or vertebral artery (surrounding vasculature) asymmetry. Therefore, the asymmetry of the surrounding vessels does not seem to be a promising morphological parameter for the evaluation of probability of rupture and growth in basilar tip aneurysms in future studies.

## Introduction

In the treatment of patients with unruptured aneurysms, the physician often faces the decision whether a surgical or interventional therapy or a watchful waiting approach should be preferred. The aim is to find the path of the lowest total risk for the patient, while comparing the individual risk of aneurysm rupture over time against the risk of the intervention. In recent years, this risk assessment of the likelihood of rupture has mainly been based on metric variables such as the height as well as the morphology of the aneurysms. [9, 11, 12, 25] Lately, studies revealed the need to assess aneurysms in a location-specific manner; hence, the known morphological parameters appear to be specific for different locations. [2, 16, 20] For example, recent studies indicated that the aneurysm size is not an adequate indicator for the probability of rupture in aneurysms of the arteria communicans anterior (ACOM). [2, 16] As a result, localization-specific factors that can influence the hemodynamics around the origin of aneurysms have come into focus. [16] The surrounding vasculature was of particular interest in this case because their anatomical changes can demonstrably influence the local flow dynamics. [3, 23] Recent studies revealed a wider bifurcation angle, including the medial cerebral artery (MCA) [1], the anterior cerebral artery (ACA) [28], and the basilar artery (BA) [29], can alternate the flow dynamics and promote the formation of local aneurysms. In basilar tip aneurysms (BTAs), an increased posterior cerebral artery (PCA) angle has been reported to be associated with the risk of aneurysm rupture. [3, 8] In general, however, there is no consensus in the literature regarding the flow dynamics around the vessels directly distal to the aneurysm, also known as daughter arteries. In fact, there are several publications that identify vessel diameters of daughter vessels as well as asymmetry in bifurcations as a risk factor for aneurysm rupture. [28, 30] In contrast, other studies could not confirm this correlation between the distal vascular diameter or asymmetries and the risk of rupture. [6, 13]

In general, aneurysms of the posterior region are associated with an increased risk of rupture [10, 15, 25] and are also significantly associated with worse clinical outcomes, independent of the treatment modality. [15] Besides, BTAs also present a unique anatomic configuration and it is well known that the supply and discharge vessels of the basilar artery are often subject to anatomical asymmetries. [7, 14, 19, 21] Nowadays, it is unclear to what extent this asymmetry of the vertebral arteries (VAs) and the PCAs affects the local flow dynamics or if it is a risk factor for aneurysm rupture or aneurysm growth in BTAs.

The present study aims to elucidate a potential association between the asymmetry of the PCAs and the VAs with the development and size of BTAs.

## Material and methods

**Fig. 1:**
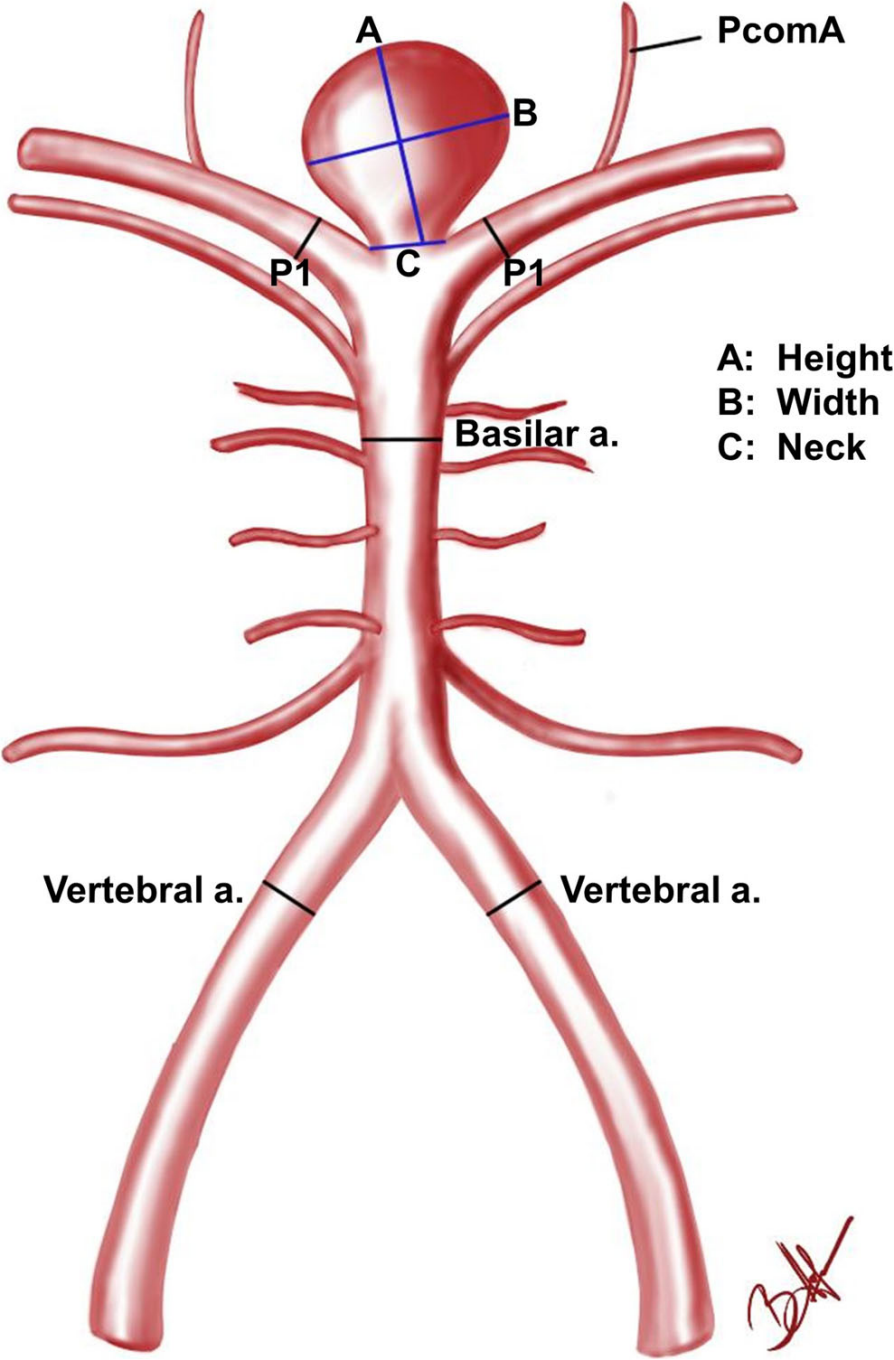
Factors measured in the study. In the study the diameters of the basilar artery, vertebral arteries, P1 arteries, aneurysm neck, height and width as well as bottleneck (width/neck), size (height/width) and aspect ratio (height/neck) and the aneurysm angle to the basilar artery were examined. PcomA. posterior communicating artery; P1. pre-communicative segment of the posterior cerebral artery

This retrospective study was approved by the local ethics committee (Nr.: 2019-820). We analyzed the CT angiographies (CTAs) of patients with basilar aneurysms admitted to our hospital during the years 2006–2019. As controls, patients with no aneurysms were chosen who had either a CT angiography during a mild head trauma or patients with aneurysms in the anterior circle of Willis. The CTAs were analyzed by a younger and a senior neurosurgeon (LL and AKP). The parameters included in the study are as follows: diameters of left and right vertebral arteries (4 mm before basilar artery origin), diameters of left and right P1 arteries (from basilar artery bifurcation to PcomA-PCA juncture; 2 mm after basilar artery bifurcation), basilar artery diameter 4 mm before the bifurcation, aneurysm lobulation, basilar aneurysm parameters (maximum height, width and neck size, ratios of width to height, height to neck, width to neck), maximum angulation of the aneurysm to basilar artery in sagittal or coronal sections of the CTA, and the ratio of smaller P1 diameter to basilar artery diameter (Fig. 1). The CTAs analyzed in cases of ruptured aneurysms were performed 4–12 h after rupture. For the purpose of this study, WFNS and Fisher grades were unnecessary and not included. Only basilar tip aneurysms were included.

Three groups of patients were compared. One group included patients without aneurysms or aneurysms in the anterior circle of Willis. These control patients received CTA to rule out an aneurysm, e.g., in the case of persistent migraines, unexplained syncope, and mild TBI with no signs of brain swelling. Conversely, severe traumatic brain injuries were not used as control patients in order to rule out a change in anatomy due to swelling. This control group is divided into two groups in order to distinguish between patients with no aneurysms and those with unrelated aneurysms of the posterior circulation. The second group consisted of patients with unruptured basilar tip aneurysms and the third group consisted of patients with ruptured basilar tip aneurysms.

For both the vertebral and P1 arteries, the diameter of the smaller artery was divided by the diameter of the larger artery for every patient and named Vertebral-ratio and P1-ratio, respectively, representing the vertebral vessel asymmetry and the P1 vessel asymmetry. As part of the analysis, the patients and subgroups were dichotomized into patients with a P1 ratio less than the mean P1 ratio of the control patients (representing a high asymmetry) versus patients with a P1 ratio greater than the mean P1 ratio of the control patients (representing a low asymmetry). Data from all control patients (patients without aneurysms and patients with aneurysms in the anterior circle of Willis) were used in the calculation of the mean P1 ratio.

Statistical analysis was performed by our department`s mathematician (IF). Groups were compared using two-sample Student’s *t* test, if both groups were normally distributed or consisted of over 30 observations. Otherwise, the Wilcoxon rank sum test was used. The group normality was tested using the Shapiro-Wilk test. A Bonferroni-corrected significance level of 0.05 was used. A Wilcoxon signed rank test was performed to ascertain the difference in size ratios. All computations were performed in R, version 3.6.1 (R Core Team (2019) (R: A language and environment for statistical computing. R Foundation for Statistical Computing, Vienna, Austria. URL https://www.R-project.org/.)

## Results

### Demographics

We studied the CTAs of 183 patients. One hundred patients served as the control group (50 without aneurysms and 50 with aneurysms of the anterior circle of Willis). Twenty-seven patients had unruptured basilar tip aneurysms and 56 had ruptured basilar tip aneurysms.

The mean age of the control group was 57 years (SD ± 16 years) without aneurysms and 57 years (SD ± 14.6 years of age) with aneurysms in the ant. circle of Willis. Sixty-nine patients were female and 31 were male.

In the groups of patients with ruptured basilar tip aneurysms, the mean age was 67 years (SD ± 11 years of age) with 22 female and 5 male patients. In the group of unruptured basilar tip aneurysms, the mean age was 67 years (SD ± 17 years) with 33 female and 23 male patients. Twenty-two patients had a second aneurysm apart from the basilar tip.

The patient age in the control group was 10 years younger than the study groups for basilar tip aneurysms. However, there was no difference in concomitant factors of vascular pathologies, such as hypertension or nicotine abuse.

### Diameter of the vessels of the posterior circulation

Table 1 gives an overview of the vessel diameter measured in each group. There was no significant difference in the diameter of the left and right side vertebral arteries or P1 arteries and no significant difference of P1, vertebral, and basilar arteries in the four groups. Also, there was no significant difference in vessel diameter when comparing gender or age.

**Table 1:** Descriptive table of mean vessel size in mm

Groups	Vertebral A. left		Vertebral A. right		Basilar A.		P1 left		P1 right	
BTA ruptured	Sum: 2.97		Sum: 2.37		Sum: 3.46		Sum: 1.90		Sum: 1.88	
	m: 3.22	f: 2.91	m: 2.14	f: 2.43	m: 3.46	f: 3.46	m: 1.78	f: 1.92	m: 1.98	f: 1.85
	< 65: 3.08	> 65: 2.83	< 65: 2.33	> 65: 2.43	< 65: 3.45	> 65: 3.48	< 65: 1.96	> 65: 1.82	< 65: 1.99	> 65: 1.73
BTA unruptured	Sum: 2.63		Sum: 2.46		Sum: 3.31		Sum: 1.75		Sum: 1.82	
	m: 2.80	f: 2.52	m: 2.58	f: 2.38	m: 3.53	f: 3.15	m: 1.79	f: 1.72	m: 1.94	f: 1.75
	< 65: 2.29	> 65: 2.93	< 65: 2.12	> 65: 2.76	< 65: 2.78	> 65: 3.77	< 65: 1.63	> 65: 1.85	< 65: 1.70	> 65: 1.94
Ant. circ. aneurysm (control group)	Sum: 2.46		Sum: 1.97		Sum: 2.74		Sum: 1.40		Sum: 1.29	
	m: 2.24	f: 2.50	m: 2.11	f: 1.94	m: 2.68	f: 2.75	m: 1.41	f: 1.39	m: 1.41	f: 1.39
	< 65: 2.45	> 65: 2.48	< 65: 2.06	> 65: 1.76	< 65: 2.74	> 65: 2.73	< 65: 1.39	> 65: 1.41	< 65: 1.39	> 65: 1.41
No aneurysm (control group)	Sum: 2.66		Sum: 2.35		Sum: 2.87		Sum: 1.43		Sum: 1.43	
	m: 2.88	f: 2.47	m: 2.47	f: 2.25	m: 3.03	f: 2.73	m: 1.5	f : 1.38	m: 1.56	f: 1.32
	< 65: 2.53	>65: 2.91	< 65: 2.29	> 65: 2.46	< 65: 2.77	> 65: 3.06	< 65: 1.40	> 65: 1.51	< 65: 1.40	> 65: 1.49

There was no statistically significant difference in the diameter of vessels between the 4 groups. There was no significant difference in mean vessel size between males and females and individuals aged > 65 years and < 65 years in each subgroup. *m*, mean diameter in mm for all male patients of this subgroup; *f*, mean diameter in mm for all female patients of this subgroup; *< 65*, mean diameter in mm for all patients under the age of 65 patients of this subgroup; *> 65*, mean diameter in mm for all patients over the age of 65 patients of this subgroup); *A.*, artery; *Ant. circ.*, anterior circulation; *BTA*, basilar tip aneurysm; *P1*, pre-communicative segment of the posterior cerebral artery

### Vertebral- and P1-ratio

**Fig. 2:**
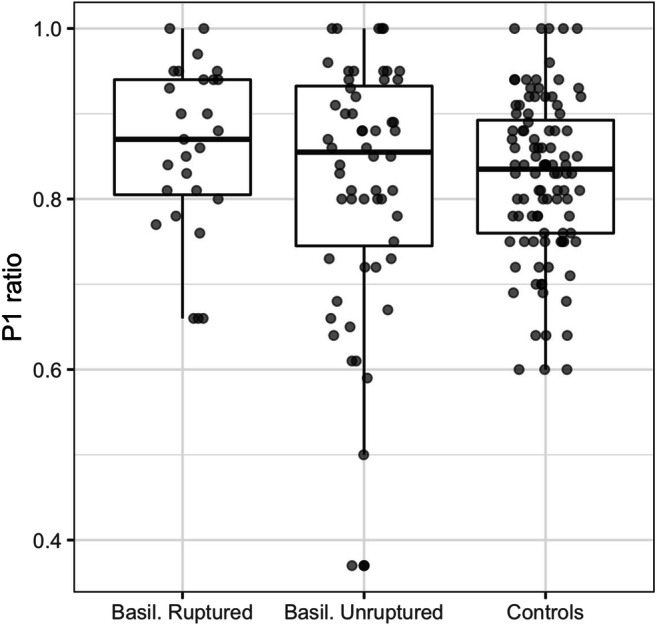
P1-ratio in basilar tip aneurysms. Boxplot diagram showing the P1 asymmetry ratios for ruptured and unruptured basilar tip aneurysms and a control group with no aneurysms or aneurysms of the anterior circulation. The differences are not statistically significant. P1, pre-communicative segment of the posterior cerebral artery

A possible implication of vertebral vessel asymmetry and P1 asymmetry in relation to development and/or rupture of basilar tip aneurysms was assessed. Statistical analysis of the Vertebral-ratio and P1-ratio showed no significant difference between the ratios of the patients with a ruptured BTA and unruptured BTA and the control group. Table 2 and Fig. 2 show the mean ratios for each individual group. There was no significant difference in the Vertebral-ratio between the ruptured BTA and the control group (*p* = 0.853), between unruptured BTA and the control group (*p* = 0.720), and between controls with and without aneurysms (*p* = 0.408). The same applied to the P1-ratio of the same groups (*p* = 0.061, *p* = 0.703, *p* = 0.993). Furthermore, there was no difference between the groups for a ratio of the diameter of the smallest P1 and the diameter of the basilar artery (data not shown).

**Table 2:** Mean small/big ratio of vertebral and P1 arteries

Groups	Vertebral-ratio	P1-ratio
BTA ruptured	0.733	0.86
BTA unruptured	0.741	0.825
Ant. circ. aneurysm	0.738	0.821
No aneurysm	0.768	0.829

The Vertebral-ratio and the P1-ratio are given in the table. No significant difference could be found between each group. There is no significant difference for the Vertebral-ratio between ruptured BTA and the whole control group (*p* = 0.853), unruptured BTA and the whole control group (*p* = 0.720), and control group with aneurysms and control group without aneurysm (*p* = 0.408). The same applies to the P1-ratio of the respective groups (*p* = 0.061, *p* = 0.703, *p* = 0.993). *Ant. circ.*, anterior circulation; *BTA*, basilar tip aneurysm

### Anatomy in ruptured and unruptured basilar tip aneurysms

A closer look between the ruptured and unruptured basilar tip aneurysms was taken to ascertain whether anatomical factors of the aneurysm and the P1 asymmetry are a risk factor for ruptured aneurysms. As Table 3 shows, the height, width, and neck of the ruptured aneurysms were greater than in the unruptured group, whereas width (*p* = 0.029) and neck (*p* = 0.004) differed significantly, while there was only a trend for the height (*p* 0.074). In addition, there was no statistically significant difference in terms of bottleneck (*p* = 0.799), height/width (*p* = 0.525), or height/neck (*p* = 0.841) aspect ratio. The P1-ratio was not statistically significant between the two groups (*p* = 0.365).

**Table 3:** Characteristics between ruptured and unruptured basilar tip aneurysms

Groups	Basilar A. diameter (mm)	Height (mm)	Width (mm)	Neck (mm)	Angle in °	Width/neck “bottleneck”	Height/width ratio	Hheight/neck ratio	P1-ratio
BTA ruptured	3.46	10.9	10.2*	4.88**	146.8	1.84	1.23	2.11	0.860
BTA unruptured	3.31	8.92	7.14*	3.73**	153.4	1.61	1.30	2.07	0.825

Basilar A. diameter, height, width, neck, angle, width/neck ratio, height/width ratio, height/neck ratio, and P1-ratio for ruptured and unruptured BTA. Significance was defined as *p* values < 0.05. Significant difference between the two groups for “width” (*p = 0.029) and “neck” (**p = 0.004) and a trend for “height” (*p* = 0.074) could be observed. No significant difference between the groups for the mean “angle” (*p* = 0.148), “bottleneck” (*p* = 0.799), “height/width ratio” (*p* = 0.5249), “height/neck ratio” (*p* = 0.841), and “P1-ratio” (*p* = 0.365). *A.*, artery; *BTA*, basilar tip aneurysm; *P1*, pre-communicative segment of the posterior cerebral artery

### P1 ratio is unrelated to aneurysm size

In order to characterize the aneurysm anatomy in relation to P1 asymmetry in the ruptured aneurysm group, the group was dichotomized into patients with high and low P1 asymmetry. There was no difference in the aneurysm characteristics between the two groups (Table 4). The same subgroup analysis was performed for the unruptured basilar tip aneurysm group, as shown in Table 5. For this group, there was also no significant relation between aneurysm size and P1 asymmetry (Table 5).

**Table 4:** Characteristics of ruptured basilar tip aneurysms

Ruptured BTA	Basilar A. diameter (mm)	Height (mm)	Width (mm)	Neck (mm)	Width/neck “bottleneck”	Height/width ratio	Height/neck ratio
High P1 asymmetry	3.19	11.2	9.67	4.43	2.15	1.24	2.48
Low P1 asymmetry	3.6	10.7	10.5	5.13	1.66	1.23	1.90

High/low P1 asymmetry as described in “Material and methods.” Significance was defined as *p* values < 0.05. No significant difference for the listed aneurysm characteristics could be observed (“Basilar A. diameter”: *p* = 0.19; “height”: *p* = 0.87; “width”: *p* = 0.80; “neck”: *p* = 0.42; “bottleneck”: *p* = 0.35; “height/width ratio”: *p* = 0.93; “height/neck ratio”: *p* = 0.19). *A.*, artery; *BTA*, basilar tip aneurysm; *P1*, pre-communicative segment of the posterior cerebral artery

**Table 5:** Characteristics of unruptured basilar tip aneurysms

Unruptured BTA	Basilar A. diameter (mm)	Height (mm)	Width (mm)	Neck (mm)	Width/neck “bottleneck”	Height/width ratio	Height/neck ratio
High P1 asymmetry	3.60	9.78	7.80	3.89	1.61	1.33	2.24
Low P1 asymmetry	3.08	8.28	6.65	3.61	1.62	1.27	1.94

High/low P1 asymmetry as described in “Material and methods.” Significance was defined as *p* values < 0.05. No significant difference for the listed aneurysm characteristics could be observed (“Basilar A. diameter”: *p* = 0.11; “height”: *p* = 0.50; “width”: *p* = 0.47; “neck”: *p* = 0.71; “bottleneck”: *p* = 0.94; “height/width ratio”: *p* = 0.58; “height/neck ratio”: *p* = 0.26). *A.*, artery; *BTA*, basilar tip aneurysm; *P1*, pre-communicative segment of the posterior cerebral artery

### P1 asymmetry does not induce aneurysm lobulation

A possible correlation between P1 asymmetry and the aneurysm lobulation as an indirect sign for rupture risk was examined. P1 ratios were dichotomized into < mean P1 ratio (representing a high asymmetry) and > mean P1 (representing a low asymmetry), as previously described. The P1 ratio did not significantly correlate with the number of lobuli present in the aneurysm, nor the presence of additional aneurysms besides the BTA in the affected patients. This statement applies to all patients with BTAs (Table 6; subgroup 1) (*p* = 0.121), as well as the subgroup of patients with ruptured (Table 6; subgroup 2) (*p* = 0.198) and unruptured (Table 6; subgroup 3) (*p* = 0.484) BTAs.

**Table 6:** Relation between all, ruptured only, and unruptured only BTA and the number of lobuli and additional aneurysms

	Number of lobuli	Number of patients with additional aneurysms
All BTA (*N* = 83)		
High P1 asymmetry	0.303	7
Low P1 asymmetry	0.673	15
Ruptured BTA only (*N* = 56)		
High P1 asymmetry	0.444	3
Low P1 asymmetry	1.111	7
Unruptured BTA only (*N* = 27)		
High P1 asymmetry	0.25	4
Low P1 asymmetry	0.419	8

Relation between P1 asymmetry, lobulation, and number of patients with additional aneurysms in basilar tip aneurysms for “all BTAs combined,” “ruptured BTAs only,” and “unruptured BTAs only”. No significant difference in respect to lobulation could be observed; *p* = 0.121 for all BTAs; *p* = 0.198 for ruptured BTAs only; *p* = 0.484 for unruptured BTAs only; high/low P1 asymmetry as described in “Material and methods.” *BTA*, basilar tip aneurysm; *P1*, pre-communicative segment of the posterior cerebral artery

## Discussion

Prior studies have revealed the importance of including not only the morphological parameters of aneurysms but also other factors such as the surrounding vascular anatomy to evaluate the individual risk of aneurysm rupture. However, these studies have not focused on the surrounding vasculature asymmetries in BTAs and their correlation with aneurysm growth or rupture. In this study, we evaluated not only the standard morphological parameters but also to what extent asymmetries in P1 and VAs are associated with BTA size and rupture.

We saw no significant difference in aneurysm size, rupture, or lobulation associated with P1 or VA asymmetries. These findings supplement our previous data for MCA aneurysms or patients with multiple aneurysms, for which we saw no significant correlation between asymmetry of the aneurysm surrounding vasculature with the risk of rupture. [6, 13]

Other researchers present data supporting the fact that asymmetrical distal vessels (daughter vessels) influence the flow dynamics in the area of a bifurcation. [4, 17] As a result, it could be assumed that this is associated with a higher incidence of aneurysm development at the affected bifurcations. To further support this, Zhang et al. demonstrated in 2019 that asymmetric MCA bifurcations are more susceptible to the development of aneurysms. [30] Other studies were also able to correlate asymmetry of the surrounding vasculature with the incidence of aneurysms. [22, 26] However, these studies not only had a relatively small number of cases, but only aneurysms of the anterior circulatory system were examined. To our knowledge, there is still no study to date that examined the influence of asymmetry on aneurysms of the posterior circulation. As no morphological difference in the characteristics of the BTAs between a small or a large asymmetry of P1 could be observed in our study (Table 4, Table 5), it seems as if the results mentioned above cannot simply be transferred to BTAs.

An important point to consider is that the anterior and posterior circulatory system arise from different embryologic origins. As Frösen and Joutel describe in detail in their review in 2018, the ACI and the anterior circulation arise from the neural crest, whereas the posterior circulation which develops at a later stage arises from the paraxial mesoderm. [5] Therefore, hemodynamic factors are probably likely to affect vessel wall remodelling and progression of an aneurysm differently in the anterior and posterior circulations. [5] Furthermore, our data shows that there is no significant difference in the asymmetry of P1 or the VAs between ruptured and unruptured BTAs, indicating that P1 or VAs asymmetry is not a risk factor for rupture in BTAs. In contrast to this, other research groups independently observed a correlation between the risk of rupture and the asymmetry of the surrounding vasculature, most often regarding the diameter of the distal vessels. [26, 27, 30] Again, this data relies on examination of aneurysms of the anterior circulatory system. Moreover, it should be mentioned that Yifei et al. did not directly examine ruptured aneurysms in 2019, but estimated the risk of rupture by the presence of high-risk factors, such as the presence of aneurysm lobuli. [26] In contrast to these studies, our previous data with no observed correlation between daughter vessel diameter and risk of rupture for MCA mirror bifurcation aneurysms and patients with multiple aneurysms supports the findings of the present study. [6, 13]

Another factor **s**trengthening the previously mentioned data of this study is the lack of correlation between the P1 asymmetry and the number of lobuli in the BTAs. After assessment by the UCAS study and others, it is widely accepted that irregularity in shape, especially the presence of lobuli in the aneurysm, is an indirect sign for rupture risk. [16, 24] Seeing no correlation between the number of lobuli in the BTAs and the P1 asymmetry in this study, a correlation between P1 asymmetry on the rupture risk becomes even less likely (Table 6). Apart from this observation, we found no statistically significant differences in the aneurysm angulation between the ruptured or unruptured BTAs. This is contrary to the findings of other studies, attributing the inclination angle as a significant risk for aneurysm rupture. [18] Yet, it must be mentioned that only aneurysms of the anterior circulation were assessed in this study. [18]

The results of our study show that the mean angle of the aneurysm to the basilar artery, the asymmetry of the VAs, and the asymmetry of P1s do not differ in ruptured and unruptured BTAs. Since it could be shown in this study that the investigated morphological parameters of the surrounding vasculature have no correlation with the size of the aneurysm, nor differ when looking at ruptured versus unruptured BTAs, which is contrary to published data regarding aneurysms at different locations, it becomes apparent that the influence of the surrounding vasculature of aneurysms needs to be further assessed in a location-specific manner. The results of this study help to narrow down the variables to be examined by future studies. Therefore, future studies can focus primarily on more promising morphological factors, such as the PCA angle, which is proven to be associated with the rupture probability of BTAs. [3, 8]

However, some limitations are worth noting. This study is cross-sectional design and does not provide any information about longitudinal data of the aneurysms. It is unclear whether found or restored correlations in vascular architecture changes preceded the formation or rupture of the aneurysm. Therefore, no direct conclusions can be drawn about the risk of rupture. However, the lack of difference in P1 asymmetry in ruptured versus unruptured BTAs, as well the lack of correlation between P1 asymmetry and lobulation of the aneurysm, a known risk factor for rupture, suggests that P1 asymmetry does not influence the risk of rupture. Although we did study patient-specific characteristics, the retrospective study design may lead to confounding parameters. For example, the younger age in the control group could independently influence factors such as the basilar angle. Despite this, we tried to rule out a lot of confounders by using two control groups, one with aneurysms at different locations. Diameters of the vessels were measured in 2D images, which may result in inaccuracy. Moreover, complex fluid dynamics or mechanisms underlying aneurysm growth or rupture could not be explained using simplified morphological variables. Further detailed examinations of the fluid dynamics are needed, as well as validation of our findings by larger prospective follow-up studies.

One of the strengths of this study is that the measured variables are not affected by rupture of the aneurysm because they refer to the surrounding vasculature and not to the aneurysm morphology itself, as many other correlated studies do.

## Conclusions

In our study, we show that P1 asymmetry does not significantly correlate with size in BTAs, does not differ between ruptured and unruptured BTAs, and does not correlate with the number of lobuli present in BTAs. The diameter of the VAs and P1 neither correlated with the prevalence of BTAs nor differed comparing ruptured and unruptured BTAs. Contrary to findings for cerebral aneurysms in different locations, asymmetry of P1 seems to have little effect on the BTAs. This highlights that assessment not only of the aneurysm morphology itself but also of the surrounding vasculature needs to be pursued in a location-specific manner in order to ultimately build reliable data for the evaluation of the aneurysm rupture risk and the best treatment strategy for the individual patient.
